# Large-Area Nanostructure Fabrication with a 75 nm Half-Pitch Using Deep-UV Flat-Top Laser Interference Lithography

**DOI:** 10.3390/s25185906

**Published:** 2025-09-21

**Authors:** Kexin Jiang, Mingliang Xie, Zhe Tang, Xiren Zhang, Dongxu Yang

**Affiliations:** 1 School of Optoelectronic Science and Engineering, University of Electronic Science and Technology of China, Chengdu 610054, China; jiangkexin@alu.uestc.edu.cn (K.J.); 202321050521@std.uestc.edu.cn (M.X.); 2 College of Materials Science and Opto-Electronic Technology, University of Chinese Academy of Sciences, Beijing 100049, China; tangzhe23@mails.ucas.ac.cn; 3 State Key Laboratory of Optical Field Manipulation Science and Technology, Institute of Optics and Electronics, Chinese Academy of Sciences, Chengdu 610209, China

**Keywords:** laser interference lithography (LIL), dual-beam interferometer, large-area patterning, beam shaper, surface-enhanced Raman spectroscopy (SERS)

## Abstract

Micro- and nanopatterning is crucial for advanced photonic, electronic, and sensing devices. Yet achieving large-area periodic nanostructures with a 75 nm half-pitch on low-cost laboratory systems remains difficult, because conventional near-ultraviolet laser interference lithography (LIL) suffers from Gaussian-beam non-uniformity and a narrow exposure latitude. Here, we report a cost-effective deep-ultraviolet (DUV) dual-beam LIL system based on a 266 nm laser and diffractive flat-top beam shaping, enabling large-area patterning of periodical nanostructures. At this wavelength, a moderate half-angle can be chosen to preserve a large beam-overlap region while still delivering 150 nm period (75 nm half-pitch) structures. By independently tuning the incident angle and beam uniformity, we pattern one-dimensional (1D) gratings and two-dimensional (2D) arrays over a Ø 1.0 cm field with critical-dimension variation < 5 nm (1σ), smooth edges, and near-vertical sidewalls. As a proof of concept, we transfer a 2D pattern into Si to create non-metal-coated nanodot arrays that serve as surface-enhanced Raman spectroscopy (SERS) substrates. The arrays deliver an average enhancement factor of ~1.12 × 10^4^ with 11% intensity relative standard deviation (RSD) over 65 sampling points, a performance near the upper limit of all-dielectric SERS substrates. The proposed method overcomes the uneven hotspot distribution and complex fabrication procedures in conventional SERS substrates, enabling reliable and large-area chemical sensing. Compared to electron-beam lithography, the flat-top DUV-LIL approach offers orders-of-magnitude higher throughput at a fraction of the cost, while its centimeter-scale uniformity can be scaled to full wafers with larger beam-shaping optics. These attributes position the method as a versatile and economical route to large-area photonic metasurfaces and sensing devices.

## 1. Introduction

Micro- and nanopatterning provide a crucial means to bridge material microstructures with their macroscopic functionalities, enabling a wealth of advanced architectures and devices in the semiconductor industry [1,2,3,4], high-density data storage [5,6,7,8], quantum devices [9,10,11,12,13] and more [14,15,16]. Among scalable patterning routes, electron- and ion-beam lithography provide ultimate resolution yet are inherently serial and suffer from stitching and throughput limits on large areas [17,18,19]. Projection photolithography can reach deep sub-100 nm pitches but at formidable capital cost and process complexity [20]. In addition, hybrid electrochemical approaches such as mask electrolyte jet machining (MEJM) have also been explored for affordable batch fabrication of surface microstructure, but their feature sizes and long-range ordering remain largely at the microscale [21,22]. However, in many engineering scenarios, including sensors [23,24], photonic crystals [25], surface-enhanced Raman scattering (SERS) [26,27], structural color control [28,29,30], etc., the requirements for pattern complexity and overlay accuracy are less stringent. In these scenarios, periodic micro- and nanostructures such as gratings, hole arrays, or pillar arrays are especially desirable especially if they can be fabricated with high uniformity and at low cost over large areas [31].

To meet these requirements for uniform, large-area, and low-cost periodic patterns articulated above, laser interference lithography (LIL) is particularly attractive [32]. It delivers maskless, parallel exposure with simple optics and high throughput [31,33,34,35,36]. Depending on how the interfering beams are generated, three representative configurations are commonly used: (i) Lloyd’s mirror interferometer, which features a compact layout and good stability but suffers from geometric inflexibility and reduced fringe contrast due to unequal beam intensities [37,38]; (ii) transmission diffraction setups, which can yield high spatial frequencies and wavelength-independent pitches but rely on expensive, nanometer-accurate gratings and careful ±1 order balancing; and (iii) two-beam interferometers, offering independent control of incidence angles and facile switching between one-dimensional (1D) and two-dimensional (2D) patterns, thereby enabling large-area uniform exposure.

Representative studies (Table 1) illustrate the trade-offs across LIL layouts and wavelengths. Byun and Kim employed a 405 nm AlInGaN diode Lloyd’s-mirror system to pattern ~290 nm pitch gratings and dot arrays over 2 × 2 cm^2^ with remarkably simple optics, yet further pitch reduction is limited by the mirror-based alignment and contrast loss at large angles, which limits simultaneous shrinkage of pitch and preservation of field uniformity [39]. Park et al. pushed to deep-ultraviolet (DUV, 257 nm) in a Lloyd’s-mirror configuration, but the reported pitches remain in the several-hundred-nanometer range with only modest exposure areas, reflecting the difficulty of jointly achieving small pitch and large field in that geometry [40]. Liu et al. used 1064 nm two-beam LIL to fabricate hierarchical anti-icing microstructures, achieving application-oriented textures at high throughput, but the long wavelength naturally leads to coarse periods (~15 µm) and is not suited to sub−100 nm patterning [41]. More generally for two-beam interferometers, obtaining a smaller pitch at fixed wavelength requires a larger half-angle, resulting in a reduction in the beam-overlap region and tightens tolerances on pointing, intensity balance, and polarization [42]. As a result, achieving both small pitch and centimeter-scale uniformity imposes stringent requirements on the source and on beam shaping. Taken together, prior reports either provide centimeter-scale fields at near-UV/DUV with pitches well above 250 nm, or achieve pattern diversity/throughput at longer wavelengths. This leaves a gap for a cost-effective two-beam DUV platform that unites sub−150 nm pitch with centimeter-scale uniformity and versatile 1D/2D patterning.

Here we introduce a two-beam DUV-LIL platform operating at 266 nm and equipped with a diffractive flat-top beam shaper, which overcomes the beam-profile and overlap limitations of conventional two-beam layouts. At this wavelength, a moderate half-angle (62°) can be chosen that preserves a large beam-overlap region while still delivering a 150 nm period (75 nm half-pitch), and the flat-top irradiance further stabilizes uniformity across a Ø 1.0 cm exposure field. By independently tuning the incidence angle and irradiance uniformity, we pattern 1D gratings and 2D dot/hole/pillar arrays with critical-dimension variation < 5 nm (1σ). As a proof of concept, we transfer the 2D pattern into Si to realize metal-free SERS substrates that exhibit an average enhancement factor of ~1.12 × 10^4^ with 11% relative standard deviation (RSD) over 65 sites, highlighting the method’s large-area uniformity and practical utility. Overall, the flat-top DUV-LIL approach combines sub-100 nm resolution, centimeter-scale uniformity, and rapid 1D/2D switching on a low-cost platform, thereby closing the identified gap and enabling reliable large-area nanofabrication for photonics, metasurfaces and chemical sensing.

## 2. Experimental Methods

### 2.1. Experimental Principle

Our goal is to fabricate large-area gratings with uniform and narrow periods. We therefore track three plan-view metrics: the grating period *p*, the linewidth *CD*, and the duty cycle *f*. The linewidth *CD* is the width of the resist ridge measured in plan-view at a specified height percentage. The duty cycle is defined as f=CD/P. These quantities allow us to report how narrow the lines are and how uniformly they are reproduced over the full aperture.

#### 2.1.1. Period Control and Design Tradeoffs

For two-beam interference lithography the period is set only by

(1) $$P=\lambda /\left(2n\sin\theta \right)$$

where λ is the laser wavelength, θ is the incidence half-angle in air measured from the surface normal, and n is the refractive index of the surrounding medium (n≈1.0 in atmosphere). Therefore, a smaller period can be obtained by increasing θ or by reducing λ. However, large θ reduces the geometric overlap of the two beams at the wafer and increases sensitivity to polarization-dependent reflectance and to alignment. Reducing λ also reduces *p*, but it tightens requirements on sources, optics, and materials. In this work, we select a wavelength of 266 nm and an angle of 62°, which results in a pitch of approximately 150 nm and a half-pitch of about 75 nm. This configuration allows for the preservation of a substantial and uniform exposure field with stable fringe visibility.

#### 2.1.2. Determinants of Duty Cycle and Dose Measurement

At the resist plane, the fringe visibility is

(2) $$V=\frac{2\sqrt{I_{1}I_{2}}}{\left(I_{1}+I_{2}\right)}$$

where $I_{1}$ and $I_{2}$ are the irradiances of the two beams. The visibility is maximized when the two beams are power balanced and mutually coherent. The exposure dose is defined as the time integral of the sum of the two irradiances at the resist plane,

(3) $$E=(I_{1}+I_{2})t$$

where *t* is the exposure time. Under a positive-tone resist with a sinusoidal aerial image, the plan-view duty cycle of the retained resist lines is

(4) $$f=1-\frac{1}{\pi }\arccos\left(\frac{E_{T}/E_{avg}-1}{V}\right)$$

where $E_{avg}$ is the average dose over one period and $E_{T}$ is the dose-to-clear of the resist. This relation provides a quantitative link between dose and duty cycle at a fixed period *p*. For a fixed *p*, increasing $E_{avg}$ enlarges the cleared spaces and reduces the linewidth, so *f* decreases.

To obtain uniform duty cycle across a large field, we use a flat-top beam shaper so that both $E_{avg}$ and *V* are spatially uniform at the wafer. The wafer-plane irradiance was measured with a cosine-corrected UV irradiance head (PD300RM-UV, Ophir, Jerusalem, Israel) on a StarLite meter (NIST-traceable, Gaithersburg, MD, USA). The working dose was selected by first measuring an open-frame contrast curve to obtain the dose-to-clear and the resist contrast, followed by a small dose matrix at fixed λ and θ to tune *f* to target value while monitoring pattern quality and uniformity [49]. In this work, the shutter-gated exposure time was set as 2 s to deliver a total dose of 50 mJ cm^−2^ (here $I\approx 25\;\mathrm{mW}\;{\mathrm{cm}}^{-2}$).

### 2.2. Experimental Setup

**Figure 1:**
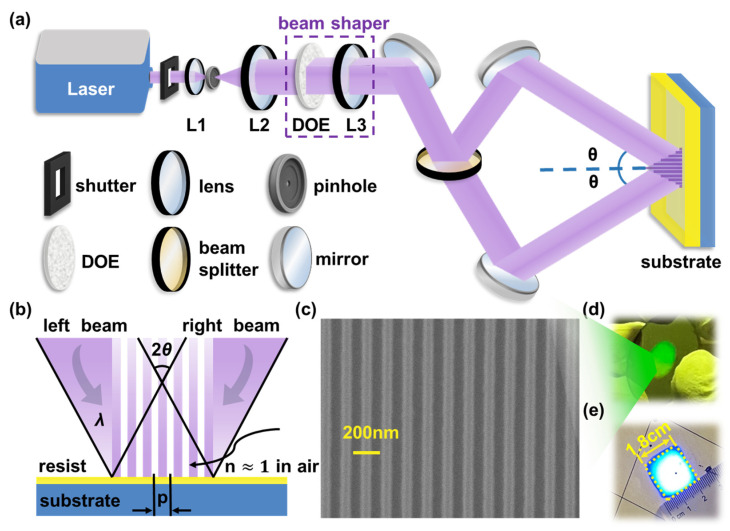
(**a**) Schematic illustration of the 266 nm deep-UV dual-beam laser interferometer. L1, L2 and L3: lenses, DOE (diffractive optical element, used together with L3 as the beam shaper), two beams impinge at symmetric half-angles θ in air (n≈1.0); (**b**) Schematic of Dual-beam laser interference lithography; (**c**) SEM top-view of a 150 nm pitch grating exposed at θ = 62°, with a wafer-plane irradiance of 30 mW·cm^−2^ (sum of both beams) for t = 1.67 s, corresponding to a total dose of D = 50 mJ·cm^−2^. Scale bar: 200 nm; (**d**) Photograph of large-area grating with a diameter of 1 cm; (**e**) Image of a reshaped flat-top beam.

**Figure A1:**
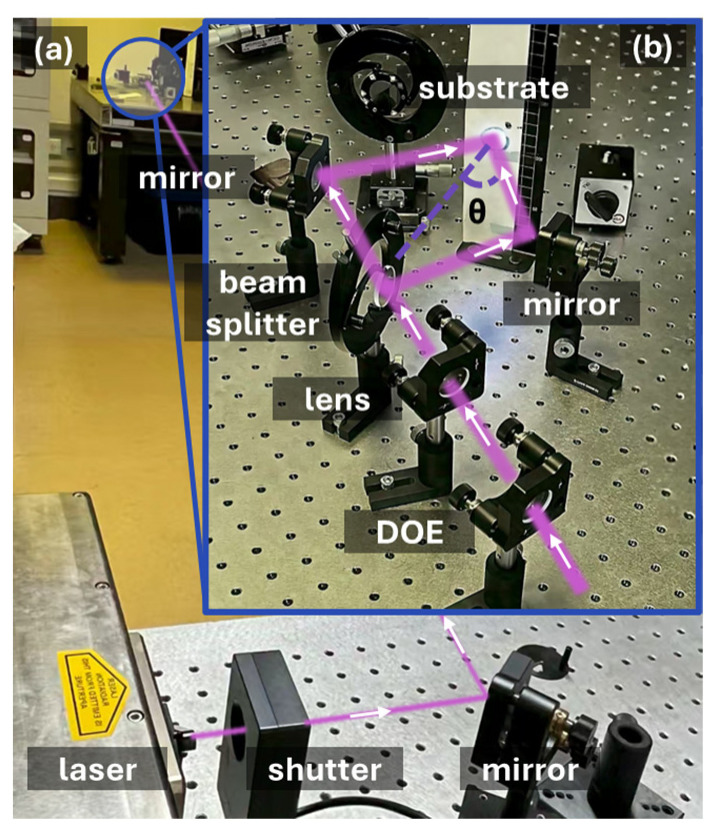
Photographs of the 266 nm dual-beam interferometer. (**a**) Source and timing section: a continuous-wave 266 nm laser followed by a fast mechanical shutter and a steering mirror; (**b**) interference section after the beam expander and pinhole: a diffractive beam shaper composed of a DOE and lens; the substrate is mounted to receive the two beams at symmetric half-angles *θ* in air.

The experimental setup of the dual-beam laser interferometer is shown schematically in Figure 1a and the physical layout on the optical table is provided in Appendix A Figure A1. A continuous-wave, 266 nm laser (FOCW 266-25, CryLaS (Berlin, Germany), 25 mW) served as the illumination source for patterning periodic 75 nm half-pitch array structures. Immediately after the laser output, a fast mechanical shutter gated the beam to set the exposure time. The laser beam with 0.58 mm diameter was firstly collimated and expanded to 5.5 mm Gaussian beam by lens 1 (L1, focal length = 30 mm) and lens 2 (L2, focal length = 301 mm), and spatially filtered by a 5 μm pinhole (P5K, Thorlabs, New Jersey, NY, USA) placed at the intermediate focus. To overcome nonuniform intensity and poor efficiency of the Gaussian beam, a beam shaper, consisting of a diffractive optical element (DOE, PT-064-W-Y-A, HOLO/OR Ltd., Ness Ziona, Israel) and a lens 3 (L3, focal length = 100 mm), was used to transform filtered Gaussian beam into a uniform-intensity flat-top beam. The flat-top beam was subsequently divided into two equal-power sub-beams by a 50R/50T UV beam splitter (BS1-266-50-1012-45UNP, CVI Laser Optics, Albuquerque, NM, USA). Each sub-beam was directed by Nd:YAG laser line mirrors (NB1-K04, Thorlabs, New Jersey, NY, USA) to overlap and interfere on the substrate at symmetric incidence angles. All interferometric exposures were performed in air (n≈1.0), θ denotes the half-angle in air measured from the substrate normal. This configuration produced a line-array interference pattern over a 1.8 × 1.8 cm^2^ area.

### 2.3. Wafer Preparation, Exposure, and Development

The fabrication process of large-area diffraction gratings consisted primarily of three steps: wafer preparation, interference exposure, and subsequent development. Firstly, a 1-inch silicon wafer was ultrasonically cleaned in acetone to ensure surface cleanliness. An adhesion promoter (Hexamethyl disilylamine, 440191, Sigma-Aldrich (St. Louis, MO, USA), 50 µL) was spin-coated onto the cleaned wafer at 2500 rpm for 30 s and then soft-baked at 100 °C for 1 min. To mitigate standing-wave effects, an organic bottom anti-reflective coating (BARC, PhiChem Corporation (Shanghai, China), 150 µL) was spin-coated at 2500 rpm, followed by baking at 200 °C for 2 min. Next, a home-synthesized positive-tone poly-hydroxystyrene (PHS) photoresist (150 µL), highly sensitive to 266 nm wavelength, was spin-coated onto the wafer at 3000 rpm to achieve a thickness of approximately ~150 nm, and baked at 100 °C for 90 s. Additionally, to suppress reflections from the silicon substrate and minimize vertical patterning artifacts, a reflective metal layer was selectively evaporated onto certain wafers prior to the photoresist coating.

Following preparation, samples were exposed using the dual-beam interferometer shown in Figure 1a, at an incidence angle of 62°, with a total irradiance of 25 mW·cm^−2^ (sum of both beams) and an exposure time of 2 s, corresponding to a total dose of 50 mJ·cm^−2^. After exposure, the samples underwent a post-exposure bake at 100 °C for 90 s and were subsequently developed in 0.26 N-tetramethylammonium hydroxide (TMAH) developer for 30 s, rinsed gently with deionized water, and dried under nitrogen flow. The resulting grating structures, featuring a half-pitch of approximately 75 nm, were examined by scanning electron microscopy (SEM, Apreo, FEI/Thermo Fisher Scientific (Waltham, MA, USA); accelerating voltage 10 kV; probe current 13 pA), as shown in Figure 1c. The high uniformity and large-area coverage (approximately 1 cm diameter) of the fabricated gratings are demonstrated in Figure 1d.

## 3. Results

### 3.1. Angle-Tuned Pitch Scaling for Large-Area 150 nm Patterns

**Figure 2:**
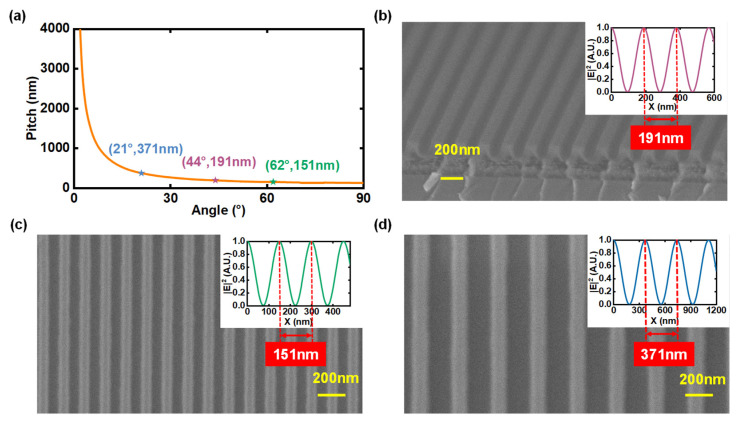
(**a**) Theoretical pitch versus interference angle at λ=266 nm with P=λ/[2sinθ], markers indicate the operating points (21°, 371 nm), (44°, 191 nm), and (62°, 151 nm); (**b**) Cross-section SEM of a P≈191 nm grating patterned at θ=44°, $E=50\;\mathrm{mJ}\;{\mathrm{cm}}^{-2}$, scale bar: 200 nm; (**c**) Cross-section SEM of a P≈151 nm grating patterned at θ=62°, $E=50\;\mathrm{mJ}\;{\mathrm{cm}}^{-2}$, scale bar: 200 nm; (**d**) Cross-section SEM of a P≈371 nm grating patterned at θ=21°, $E=50\;\mathrm{mJ}\;{\mathrm{cm}}^{-2}$, scale bar: 200 nm.

The dual-beam geometry allows straightforward pitch selection. By adjusting the relative positions of the two mirrors, as shown in Figure 1a, the incidence angle θ of the interfering beams can be precisely tuned, allowing accurate control of the grating pitch P. Figure 2a illustrates the theoretical relationship between grating pitch and incidence angle at the wavelength of 266 nm according to Equation (1). The grating pitch decreases continuously with increasing incidence angle, approaching a theoretical minimum of 133 nm at θ=90°. In practice, large angles can reduce beam overlap to less than half the flat-top diameter and make the exposure sensitive to sub-micrometer mirror drift.

Balancing resolution and fringe contrast, we chose an incidence angle of 62°, which yields a 150 nm period (75 nm half-pitch) across the full 1 cm diameter exposure area. This configuration maintains sufficient beam overlap and comfortable alignment tolerances. To verify the reliability of the setup, we also exposed samples at 44° and 21° to obtain pitches of 370 nm and 190 nm. As demonstrated by the SEM images in Figure 2c,d, the fabricated pitch values show excellent agreement with the simulated grating intensity profile in the upper-right inset and theoretical predictions in Figure 2a. Additionally, the cross-sectional SEM image presented in Figure 2b clearly demonstrates grating structures with the 190 nm sample, exhibiting near-vertical sidewalls and uniform pitch and depth, indicative of high pattern fidelity. Plan-view linewidth statistics were collected at five locations across the Ø1.0 cm field (center and four cardinal sites), with 25 lines per location (*n* = 125). The linewidth variation is within ±5 nm (1 standard deviation, s.d.) across all measured features. Low surface roughness on the grating sidewalls further confirms the high-quality patterning process, minimizing optical imperfections and enhancing device performance. Furthermore, the absence of notable structural defects, such as cracks or voids, underscores optimized fabrication conditions, ensuring structural integrity. Such precise and defect-free gratings are crucial for high-performance optical components requiring minimal wavefront distortion and maximal diffraction efficiency.

In summary, careful selection of the incidence angle enables the flat-top dual-beam system to produce centimeter-scale gratings with pitches as small as 150 nm, and the additional 190 nm and 370 nm samples corroborate the predictive model over a 2.5-fold span in pitch.

### 3.2. Uniformity Verification of Centimeter-Scale Grating Pattern

Uniformity across centimeter-scale areas is essential for reliable optical and photonic applications. Using a flat-top beam profile with dimensions of 1.8 × 1.8 cm^2^, large-area diffraction gratings with a diameter of 1 cm were successfully fabricated, as shown in Figure 1d,e. Compared with conventional Gaussian beam exposure, the flat-top beam delivers a consistent intensity distribution, significantly improving both uniformity and material utilization.

**Figure 3:**
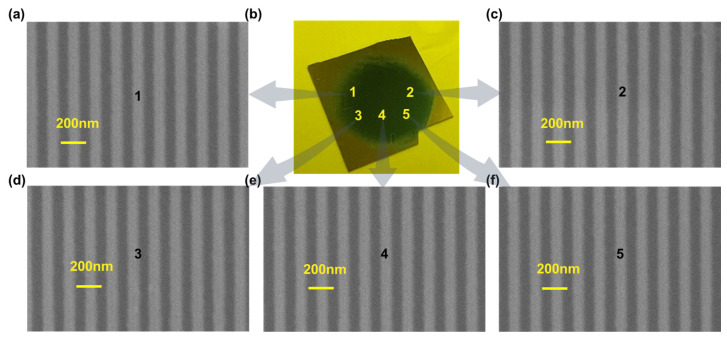
(**b**) Photograph of a centimeter-scale grating sample indicating measurement positions 1–5. (**a**,**c**–**f**) Plan-view SEM images acquired at positions 1–5. The pitch is P=156±5 nm across all sites; the top-surface line CD is 78 ± 5 nm (mean ± s.d., *n* > 100 periods per site), demonstrating high pattern uniformity.

To quantitatively evaluate pattern uniformity, five representative locations across the exposure region were analyzed (positions 1–5, Figure 3b), covering central and peripheral areas. SEM images of gratings at these positions (Figure 3a,c–f) demonstrate high spatial uniformity. Specifically, with a pitch *p* = 156 ± 5 nm (from fast Fourier transform of line scans), the top-surface line CD is 78 ± 5 nm across all sampling locations (values are mean ± s.d., *n* > 100 periods per site). Meanwhile, using ImageJ (v. 1.54p) with the 1.00 µm scale bar for pixel-to-length conversion, we measured 20 periods at the imaged site, yielding a trench depth of 114 ± 7 nm (mean ± s.d.). This low variation confirms the flat-top beam’s efficacy in mitigating uneven exposure typical of Gaussian illumination.

Achieving such precise linewidth uniformity is critical for advanced photonic devices, where even minor deviations can significantly impact performance. The measured standard deviation (5 nm) underscores the method’s repeatability and precision over large exposure areas. Additionally, improved beam uniformity reduces edge defects commonly encountered with Gaussian beams, enhancing throughput, reducing material waste, and ensuring high-yield manufacturing. Overall, the adoption of a flat-top beam in deep-UV interference lithography represents a substantial improvement for large-area patterning. Further optimization of beam shaping techniques may enable scaling this uniformity to even larger substrates, expanding applications in scalable manufacturing of high-performance photonic and optoelectronic devices.

### 3.3. SERS-Based Uniformity Assessment of Nanodot Arrays

Quantitative SERS analysis requires substrates whose electromagnetic hot spots are both densely and uniformly distributed [50]. To evaluate whether our dual-beam DUV-LIL process satisfies this criterion, we fabricated nanodot arrays by two orthogonal exposures of the photoresist, followed by reactive ion etching (RIE) of silicon to transfer the pattern into the substrate. The resist pattern acted as the etch mask during RIE, and the resist was subsequently stripped to yield Si nanodot arrays. The square lattice patterned on the pure silicon substrate generates strong electric field concentration at the lattice edges and grooves via optical diffraction and localized mode coupling, thus facilitating SERS enhancement without the involvement of conventional metallic plasmonic materials.

**Figure 4:**
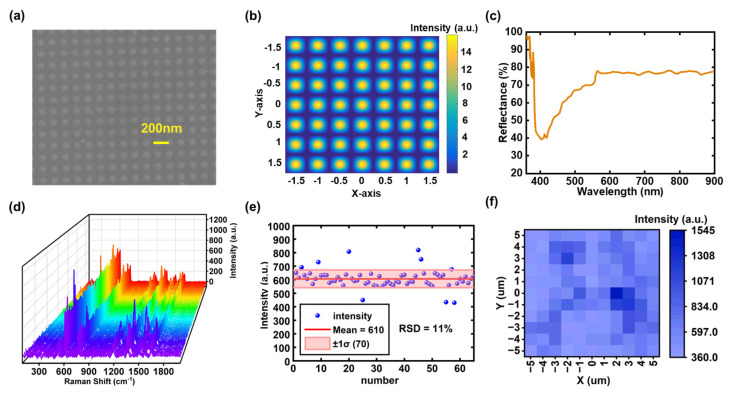
(**a**) SEM image of the fabricated nanodot array; (**b**) Simulated optical field distribution of the nanodot array structure; (**c**) Measured optical reflectance spectrum of the nanodot array. A broad minimum around 406 nm which corresponds to a resonance feature in extinction given the negligible transmission of the Si substrate. (**d**) Raman spectra of R6G molecules (10^−5^ M) measured randomly at 65 positions; (**e**) Raman intensity distribution of the 613 cm^−1^ peak measured at 65 different locations, demonstrating excellent uniformity (RSD ≈ 11%); (**f**) Raman intensity mapping of the 613 cm^−1^ peak over a 10 μm × 10 μm area.

The resulting nanodot arrays, confirmed by SEM imaging (Figure 4a) and optical simulation (Figure 4b), display a well-ordered and uniform dot arrangement. The optical reflectance spectrum (Figure 4c) exhibits a broad minimum centered at approximately 406 nm. Because the silicon substrate is non-transmissive in this spectral range, the reflectance minimum implies a resonant enhancement of the near field, indicating that the structure can support localized surface modes resonance under incident light excitation. Such efficient excitation of surface modes can significantly enhance the local electromagnetic field, which is the key factor responsible for the amplification of SERS signals.

SERS-active substrates require the SERS signals to exhibit good uniformity and reproducibility. To quantitatively evaluate the SERS activity and substrate uniformity, the substrate was immersed in a 10^−5^ M solution of Rhodamine 6G (R6G) for 30 min, followed by air-drying at room temperature. Under consistent experimental conditions, SERS spectra were collected from 65 randomly selected spots. As shown in Figure 4d, all spectra exhibit identical peak positions and line shapes. All Raman intensities are reported in arbitrary units (a.u.), as output by the spectrometer after baseline subtraction and normalization by acquisition time. The intensity distribution of the 613 cm^−1^ mode is summarized in Figure 4e, giving an average intensity $I_{mean}=610\;a.u.$ (mean over 65 spots) and a standard deviation σ=70 a.u. Consequently, the relative standard deviation is $RSD=\sigma /I_{mean}\times 100\%\approx 11\%$, and over 85% of the measured points fall within $I_{mean}\pm 1\sigma$, indicating centimeter-scale spectral uniformity of the substrate. Furthermore, a Raman mapping collected from a 10 μm × 10 μm area (Figure 4f) shows an equally uniform color distribution, confirming that the intensity homogeneity persists down to the micron scale.

The SERS enhancement factor (EF) was calculated via

(5) $$EF=\frac{I_{SERS}N_{bulk}}{I_{bulk}N_{SERS}}$$

where $I_{SERS}$ and $I_{bulk}$ are the Raman intensities at 613 cm^−1^ for the SERS substrate and the bulk solution, respectively; $N_{SERS}$ and $N_{bulk}$ are the estimated number of R6G molecules in each case. In this study, the peak intensity at 613 cm^−1^ of a 10^−5^ M R6G solution was measured to be 610 ± 70 (mean ± s.d.) a.u. across 65 randomly selected spots on the substrate. For comparison, under identical experimental conditions, the intensity of the same peak at 613 cm^−1^ was measured to be 540 ± 50 (mean ± s.d.) a.u. for a 10^−1^ M R6G solution on a bare silicon substrate. Substituting these values into Equation (5) yields an *EF* of (1.12 ± 0.13) × 10^4^ (mean ± s.d.). This value demonstrates sufficient reliability for practical detection.

In summary, the combination of effective enhancement and low signal fluctuation demonstrates that the precisely engineered periodic arrays fabricated by LIL can overcome the limitations of conventional SERS substrates, including uneven hotspot distribution and complex fabrication procedures. This, together with their centimeter-scale scalability and high throughput, highlights the potential of this technique for practical applications in ultrasensitive chemical sensing, biosensing, and on-chip integrated detection systems.

## 4. Discussion

The primary achievement of this work is the successful fabrication of periodic nanostructures with a 150 nm pitch (75 nm half-pitch) over a centimeter-scale area using a cost-effective, laboratory-scale dual-beam LIL system. While LIL is a well-established technique and the use of beam shaping is not new, our work’s specific contribution lies in the synergistic combination of a 266 nm DUV source with a diffractive flat-top beam profile. This integration addresses a critical gap identified in the existing literature, where most lab-scale systems are limited to longer wavelengths and, consequently, larger pitches, or suffer from the non-uniformity inherent to Gaussian beams. Our approach provides a scalable and accessible pathway to fabricating features well below the ~250 nm pitch typically achieved with near-UV laser sources, pushing the resolution into a regime relevant for advanced photonic and metasurface applications without resorting to prohibitively expensive industrial lithography systems.

Operating at 266 nm allows a moderate half-angle (≈62°) that preserves a large beam-overlap region while still delivering a 150 nm period, easing tolerances relative to the very large half-angles required at longer wavelengths. The use of a flat-top beam profile was instrumental in achieving the high uniformity demonstrated across the Ø1.0 cm exposure field. Conventional Gaussian beam exposures inevitably lead to dose variation from the center to the edge of the beam, resulting in a very small usable area with the correct feature dimensions. Our quantitative analysis, which revealed a critical-dimension variation of less than 5 nm across five distinct points on the sample, confirms that the flat-top beam effectively mitigates this issue. This high degree of uniformity is not merely an aesthetic improvement; it is a critical enabler for functional devices where performance depends on periodicity and consistency over a large area. The smooth, near-vertical sidewalls observed in the patterned gratings are further evidence of a well-optimized process, including the crucial use of a bottom anti-reflective coating (BARC) to suppress standing wave effects, which is essential for high-fidelity pattern transfer.

To functionally evaluate the quality and uniformity of the fabricated nanostructures, we utilized them as SERS substrates. It is important to note that the primary goal of this test was not to claim a record enhancement factor, but rather to use SERS as a highly sensitive mapping technique to probe the uniformity of the underlying response. The key challenge for the widespread adoption of SERS in quantitative chemical sensing is often poor signal reproducibility, which stems directly from an inconsistent distribution of “hot spots” on the substrate. Our results present a compelling solution to this problem. The measured relative standard deviation (RSD) of only 11% in the Raman intensity of the 613 cm^−1^ peak across 65 randomly sampled points is a direct functional confirmation of the substrate’s exceptional uniformity. This low signal variation provides strong evidence that our DUV-LIL method produces nanodot arrays with a highly ordered and consistent geometry, which in turn generates a predictable and reproducible SERS enhancement over a large area.

Under the reported conditions a Ø1.0 cm field is exposed in 2 s at a total dose of 50 mJ cm^−2^, which corresponds to an exposure-limited area rate of ~1400 cm^2^ h^−1^. For comparison, single-beam e-beam at 100 nA with 50–500 µC cm^−2^ doses writes ~1–10 cm^2^ h^−1^ for dense periodic patterns. Using the same units, the present DUV LIL therefore delivers approximately $10^{2}–10^{3}$ higher area throughput while maintaining centimeter-scale uniformity.

Looking forward, this work establishes flat-top DUV-LIL as a versatile and economical platform for nanofabrication. The same optical setup produces both one-dimensional gratings and two-dimensional dot arrays, which highlights its flexibility. The principles demonstrated here can be directly extended to the fabrication of a wide range of devices, including metasurfaces requiring sub-wavelength element spacing for operation in the visible spectrum, diffractive optics, and templates for patterned magnetic media. Future work could focus on integrating a step-and-repeat sample stage to pattern full-wafer scales, further closing the gap between academic research and industrial manufacturing needs.

## 5. Conclusions

Conventional laser interference lithography struggles to combine a 75 nm half-pitch with centimeter-scale uniformity, largely because Gaussian beam profiles narrow the exposure latitude and large half-angles reduce the usable beam-overlap region. In this study, we deliver a cost-effective two-beam deep-UV LIL platform operating at 266 nm with diffractive flat-top beam shaping that produces 150 nm period (75 nm half-pitch) gratings and 2D arrays across a Ø1.0 cm field with <5 nm (1σ) critical-dimension variation, smooth edges, and near-vertical sidewalls.

In summary, our contributions are threefold:

- **Deep-ultraviolet period setting with a moderate angle.** Using a 266 nm source and a moderate incidence half-angle, we realize 75 nm half-pitch while preserving a large beam-overlap region and stable alignment. At this wavelength the chosen angle delivers a 150 nm period with robust field coverage rather than relying on extreme angles that shrink the usable overlap.
- **Flat-top illumination for uniformity and process latitude.** A diffractive flat-top shaper combined with a relay lens produces a uniform on-wafer irradiance. Together with a power balance near unity fringe visibility, this yields centimeter-scale uniformity and tight control of duty cycle. We pattern one-dimensional gratings and two-dimensional arrays over a Ø1.0 cm field with critical-dimension variation below 5 nm (1σ), smooth edges, and near-vertical sidewalls.
- **Application validation with SERS arrays.** We validate utility using silicon-transferred 2D nanodot arrays as SERS substrates, where low signal variation across 65 randomly sampled points (11% RSD) indicates a highly ordered and consistent geometry and addresses the uneven hotspot distribution and complex fabrication procedures common to conventional SERS substrates, with an apparent enhancement factor of ~1.12 × 10^4^.

Taken together, coupling a diffractive flat-top beam with 266 nm illumination turns LIL into a practical, low-barrier route to 75 nm patterning with centimeter-scale uniformity. This capability provides an immediately useful platform for rapid prototyping and statistically robust device studies in photonics, metasurfaces and on-chip sensing without reliance on high-cost serial tools.

## Figures and Tables

**Table 1:** Comparison of Grating Period and Area in Nanofabrication Using Laser Interference Lithography.

Method	Wavelength (nm)	Pattern Type	Resolution (Pitch, nm)	Area	Ref.
Two-beam laser interferometer	266	Grating/Dot array/hole array	150	Ø1.0 cm	This paper
Two-beam laser interferometer	422	Grating	600	1 µm × 1 µm	[41]
Two-beam laser interferometer	1064	Grating	15,000	--	[43]
Lloyd’s mirror interferometer	257	Grating	770	3 cm × 3 cm	[40]
Lloyd’s mirror interferometer	325	Dot Array	478	Centimeter scale	[44]
Lloyd’s mirror interferometer	325	Grating	250	--	[45]
Lloyd’s mirror interferometer	355	Grating	600	--	[46]
Lloyd’s mirror interferometer	405	Grating	500	--	[47]
Lloyd’s mirror interferometer	405	Grating/Dot Array	290	2 cm × 2 cm	[39]
Lloyd’s mirror interferometer	442	Grating/Dot Array	1304	--	[48]
Lloyd’s mirror interferometer	785	Grating	570	--	[27]

## Data Availability

The authors confirm that the data supporting the findings of this study are available within the article.
